# Lack of associations between *LIG3* gene polymorphisms and neuroblastoma susceptibility in Chinese children

**DOI:** 10.7150/jca.33605

**Published:** 2019-10-03

**Authors:** Jiwen Cheng, Kongmei Wei, Yijuan Xin, Pu Zhao, Jiao Zhang, Wei Jia, Baijun Zheng

**Affiliations:** 1 Department of Pediatric Surgery, the Second Affiliated Hospital of Xi'an Jiaotong University, Xi'an 710004, Shaanxi, China.; 2 Department of Clinical Laboratory, LanShi Hospital of Lanzhou, Lanzhou 730050, Gansu, China.; 3 Clinical Laboratory Medicine Center of PLA, Xijing Hospital, Air Force Medical University, Xi'an 710032, Shaanxi, China.; 4 Department of Neonatology, Shaanxi Provincial People's Hospital, Xi'an 710068, Shaanxi, China.; 5 Department of Pediatric Surgery, the First Affiliated Hospital of Zhengzhou University, Zhengzhou 450052, Henan, China.; 6 Department of Pediatric Surgery, Guangzhou Institute of Pediatrics, Guangzhou Women and Children's Medical Center, Guangzhou Medical University, Guangzhou 510623, Guangdong, China.

**Keywords:** neuroblastoma, *LIG3*, polymorphism, case-control, susceptibility

## Abstract

Accumulating evidence suggests that dysregulation of the DNA non-homologous end-joining (NHEJ) repair system is a causative factor in many cancers, including high-risk neuroblastoma. A number of studies have shown that polymorphisms in the DNA *ligase III* (*LIG3*) gene, one of the key genes in the error-prone alternative NHEJ (a-NHEJ) pathway for DNA double-strand break (DSB) repair, are associated with a variety of cancers. Nevertheless, whether *LIG3* polymorphisms contribute to neuroblastoma risk remains unknown. We investigated the correlation between neuroblastoma susceptibility and two *LIG3* polymorphisms (rs1052536 C>T and rs4796030 A>C) among 469 neuroblastoma patients and 998 healthy controls from China. Our results failed to detect any relationship between the analyzed SNPs and neuroblastoma risk in either overall analysis or stratification analysis. These results suggest that rs1052536 C>T and rs4796030 A>C are unrelated to neuroblastoma susceptibility in the Chinese population. Further studies with larger sample sizes and multiple ethnicities are necessary to verify our results.

## Introduction

Neuroblastoma is one of the most common extracranial solid tumors in childhood. These tumors originate from abnormally differentiated or undifferentiated sympathetic nervous system stem cells and demonstrate highly heterogeneous biological characteristics and clinical manifestations. A minority of cases can resolve themselves, but most exhibit distant metastases at the time of diagnosis. Along with the advancement of treatment technology, the 5-year survival rate of low/medium-risk neuroblastoma has increased to more than 90%, but the 5-year survival rate of children with high-risk neuroblastoma is still less than 50% 1. Notably, approximately 60% of patients are diagnosed with high-risk neuroblastoma.

The etiology of neuroblastoma is currently unclear, but accumulating evidence implicates disorders of DNA damage repair in abnormal cell proliferation 2. In neuroblastoma, genome amplification of the *MYCN* gene encoding N-myc proto-oncogene protein (N-Myc) and segmental chromosomal alterations, including 1p heterozygous deletions, 11q heterozygous deletions, and 17q increases, are associated with tumor progression and poor prognosis 3. Pashaiefar et al. 4 found that expression of the DNA *ligase III* (*LIG3*) gene was abnormally elevated in bone marrow specimens from patients with acute myeloid leukemia. Furthermore, MYCN binds to the promoter region of *LIG3*, thereby increasing its transcriptional activity and leading to enhanced cellular tyrosine kinase activity 4. Newman et al. 5 found that DNA double-strand breakage contributes to human neuroblastoma and that the alternative (a)-NHEJ pathway involving *LIG3* is a therapeutic target for high-risk neuroblastoma. *LIG3* polymorphisms influencing expression may contribute to neuroblastoma susceptibility. In fact, Landi et al. 6 found that the *LIG3* rs1052536 single nucleotide polymorphism (SNP) was significantly associated with lung cancer risk among the young population. Moreover, genetic polymorphisms of *LIG3* are strongly related to breast cancer prognosis 7. However, whether *LIG3* polymorphisms are also associated with neuroblastoma risk remains unclear. Therefore, we conducted a three-center case-control study including 469 neuroblastoma patients and 998 controls to investigate the association between *LIG3* gene polymorphisms and neuroblastoma susceptibility in Chinese children.

## Materials and Methods

### Study subjects

A total of 469 patients with histopathologically confirmed neuroblastoma and 998 cancer-free controls were recruited from the Guangzhou Women and Children's Medical Center, The First Affiliated Hospital of Zhengzhou University, and The Second Affiliated Hospital of Xi'an Jiaotong University between December 2007 and March 2018. Cases and controls were matched according to sex, age, and ethnicity. The study was approved by the ethics committee of each institution, and informed consent was acquired from each parent or guardian. Each participant donated 2 ml of venous blood for genomic DNA extraction.

Inclusion criteria: newly diagnosed and histopathologically confirmed neuroblastoma patients; patients not received treatment other than surgery prior to peripheral blood sample collection; all cases were unrelated Han Chinese; patients under the age of 15 years.

Exclusion criteria: those who did not meet the inclusion criteria; did not have complete clinical information; did not sign the informed consent form.

### Polymorphism selection and genotyping

We screened for potentially functional polymorphic sites located in the 5' flanking region, the 5' untranslated region (UTR), the 3' UTR, and the *LIG3* exon region according to previously summarized criteria 8. Two SNPs, rs1052536 C>T and rs4796030 A>C, were selected from the NCBI dbSNP database (http://www.ncbi.nlm.nih.gov/projects/SNP) and SNPinfo (http://snpinfo.niehs.nih.gov/snpfunc.htm).

Genomic DNA was extracted from 2 mL of peripheral blood using the TIANamp Blood DNA Kit (TianGen Biotech Co. Ltd., Beijing, China) following the manufacturer's instructions. Genotyping was performed using TaqMan real-time PCR assays on the 7900 HT sequence detector system (Applied Biosystems, Foster City, CA, USA) following a published protocol 8, 9. Approximately 10% of the samples were randomly selected for repeated analysis as quality controls. The agreement rate of SNP detection in the replicate group was 100%.

### Statistical analysis

All statistical analyses were conducted using the SAS statistical analysis package (version 9.1, SAS Institute, Cary, NC). Deviation from Hardy-Weinberg equilibrium (HWE) was assessed among control subjects by the goodness-of-fit χ^2^ test. Demographic and allele frequency differences between the case and control groups were evaluated using a two-sided χ^2^ test. The association between *LIG3* gene polymorphisms and neuroblastoma susceptibility was analyzed using a logistic regression model. We also performed a hierarchical analysis of *LIG3* polymorphisms stratified by age, sex, tumor locations, and clinical stages. Values of *P*<0.05 were considered statistically significant for all tests.

## Results

### Correlation of *LIG3* gene polymorphisms with neuroblastoma susceptibility

The demographic characteristics for combined subjects and Shaanxi subjects can be found in **Supplementary Table S1**. **Table 1** summarizes the genotype frequencies of *LIG3* rs1052536 and rs4796030 polymorphisms and the associations of these SNPs with neuroblastoma susceptibility. Neither polymorphism deviated from HWE (*P* values of 0.902 and 0.478, respectively). In the control group, there is no significant difference between the actual distribution of the above polymorphisms and the theoretical distribution, which satisfies the population genetics law and is well represented.

**Table 2** summarizes the stratification analyses for patient age, sex, tumor origins, and clinical stages. Again, there were no significant associations between *LIG3* gene polymorphisms and neuroblastoma susceptibility in any subgroup (*P*>0.05).

## Discussion

DNA double-strand breaks (DSBs) are the most severe form of damage to the integrity and stability of the genome. Failure to repair this damage or errors in repair can lead to genome instability and abnormalities in cell proliferation. The main DSB repair mechanisms in mammalian cells include the homologous recombination (HR) repair and non-homologous end-joining (NHEJ) repair pathways. NHEJ is divided into two pathways: classical NHEJ (c-NHEJ) and a-NHEJ 10. There is accumulating evidence that NHEJ dysregulation is a causative factor in many cancers, such as neuroblastoma 5, breast cancer 7, chronic myeloid leukemia 11, lung cancer 6, esophageal squamous cell carcinoma 12, pancreatic cancer 13, and non-oral or squamous cell carcinoma 14. The a-NHEJ pathway participates in binding, cleavage, and ligation of DNA breaks and depends on PARP1, the MRN complex, LIG1, and LIG3, excluding several key genes of the c-NHEJ pathway. Compared to c-NHEJ, a-NHEJ is more prone to false repairs, with missing or unbalanced translocations. Chromosomal translocation is a driving factor for tumorigenesis in a variety of cell types, and the error-prone repair characteristics of a-NHEJ make it a key factor for genomic instability and tumorigenesis 15.

The human *LIG3* gene is located on chromosome 17 q11.2-q12 16. Unlike *LIG1* and *LIG4*, *LIG3* encodes three or four different DNA ligase polypeptides (including DNA ligases IIIα and IIIβ) with distinct functions in amphibians and mammals 17. Among them, nuclear Lig IIIα is mainly involved in excision repair and a-NHEJ repair 18, and mitochondrial Lig IIIα is involved in mitochondrial genome replication and repair 19. Furthermore, Lig IIIα is frequently overexpressed in cancer cells and is a biomarker for increased dependence upon a-NHEJ for DSB repair 17. It is also considered a potential novel target for tumor therapy 20.

A growing body of research indicates that *LIG3* SNPs are significantly associated with cancer susceptibility and recurrence 5-7, 11-14. Landi et al. 6 found that *LIG3* rs1052536 is significantly associated with susceptibility to lung cancer among young people. Surprisingly, breast cancer patients with *LIG3* rs1052536 tend to demonstrate longer progression-free survival 7. Zhu et al. 14 reported that *LIG3* rs4796030 can significantly modify the recurrence risk of non-oropharyngeal squamous cell carcinoma. Other *LIG3* SNPs are also significantly associated with susceptibility to esophageal squamous cell carcinoma and pancreatic cancer 12, 13. Daniele et al. 21 reported that multiple myeloma patients with elevated expression of *LIG3* mRNA tend to have a worse prognosis, and *LIG3* mRNA expression was elevated during disease progression and relapse. They also found that *LIG3* knockdown significantly increased DNA damage and ultimately inhibited the growth of multiple myeloma cells *in vivo* and *vitro*. In the current study, we comprehensively assessed the associations between *LIG3* polymorphisms and neuroblastoma risk but did not detect a significant association. Erika et al. 5 found that the expression levels of a-NHEJ proteins, such as LIG3, LIG1, and PARP1, were significantly upregulated in high-risk neuroblastoma cells compared to those in normal cells. Reduction in LIG3 and LIG1 expression levels in neuroblastoma cells led to DSB accumulation and cell death. In addition, they also found that patients with high expression of LIG3 in neuroblastoma cells had poorer prognoses. Thus, LIG3 may have a tumor-promoting role in neuroblastoma. Since *LIG3* SNPs can affect the expression and activity of *LIG3*, we speculate that neuroblastoma patients with *LIG3* SNPs may have longer overall survival and (or) tumor-free survival than those with higher expression of *LIG3* mRNA or without these SNPs, although this speculation needs confirmation. If this hypothesis can be further validated, the use of mutant genes to induce *LIG3* loss-of-function may be useful for the treatment of high-risk neuroblastoma patients.

This is the first study to assess the associations between *LIG3* SNPs (rs1052536 and rs4796030) and susceptibility to neuroblastoma in Chinese children; however, there are several limitations. First, this study is retrospective and may be limited by information and selection bias. Neuroblastoma is a heterogeneous tumor with a complex etiology, and we failed to obtain information on potentially important environmental factors, such as living environment, diet, and pregnancy exposure in this study. These factors should be addressed in the future. Second, despite the relatively large total sample sizes included in the study, some subgroup sample sizes were relatively small, which inevitably diminished the statistical power. Third, only two polymorphisms in the *LIG3* gene were studied. Other potentially functional *LIG3* polymorphisms may also modify the encoded helicase or the activity of gene and thus should be involved in further study. Fourth, whether *LIG3* SNPs are closely related to prognosis of neuroblastoma was not studied in this study, which needs to be studied further. Finally, because the subjects were mainly from the Han population in different parts of China, the conclusions drawn may not be directly applicable to people of other ethnicities.

In conclusion, these results do not support a significant association between *LIG3* gene polymorphisms and susceptibility to neuroblastoma; it is necessary to expand the sample size and conduct epidemiological studies on additional polymorphic loci in different ethnic groups.

## Supplementary Material

Supplementary figures and tables.

## Figures and Tables

**Table 1:** Associations between *LIG3* gene polymorphisms and neuroblastoma susceptibility

Genotype	Cases (N=469)	Controls (N=998)	*P*^ a^	Crude OR (95% CI)	*P*	Adjusted OR (95% CI) ^b^	*P*^ b^
**rs1052536 (HWE=0.902)**							
CC	224 (47.76)	479 (48.00)		1.00		1.00	
CT	199 (42.43)	426 (42.69)		1.00 (0.79-1.26)	0.993	1.00 (0.79-1.26)	0.993
TT	46 (9.81)	93 (9.32)		1.06 (0.72-1.56)	0.775	1.06 (0.72-1.56)	0.773
Additive			0.956	1.02 (0.86-1.20)	0.843	1.02 (0.86-1.20)	0.833
Dominant	245 (52.24)	519 (52.00)	0.933	1.01 (0.81-1.26)	0.933	1.01 (0.81-1.26)	0.920
Recessive	423 (90.19)	905 (90.68)	0.765	1.06 (0.73-1.54)	0.764	1.06 (0.73-1.54)	0.765
**rs4796030 (HWE=0.478)**							
AA	144 (30.70)	307 (30.76)		1.00		1.00	
AC	234 (49.89)	483 (48.40)		1.03 (0.80-1.33)	0.802	1.04 (0.80-1.33)	0.790
CC	91 (19.40)	208 (20.84)		0.93 (0.68-1.28)	0.666	0.93 (0.68-1.28)	0.668
Additive			0.790	0.97 (0.83-1.14)	0.728	0.97 (0.83-1.14)	0.731
Dominant	325 (69.30)	691 (69.24)	0.982	1.00 (0.79-1.27)	0.982	1.00 (0.79-1.27)	0.973
Recessive	378 (80.60)	790 (79.16)	0.524	0.91 (0.69-1.20)	0.524	0.91 (0.69-1.20)	0.520
**Combine risk genotypes ^c^**							
0	45 (9.59)	116 (11.62)		1.00		1.00	
1	424 (90.41)	881 (88.28)		1.24 (0.86-1.78)	0.245	1.24 (0.86-1.79)	0.242
2	0 (0.00)	1 (0.10)	0.401	/	0.981	/	0.981
0	45 (9.59)	116 (11.62)		1.00		1.00	
1-2	424 (90.41)	882 (88.38)	0.246	1.24 (0.86-1.78)	0.247	1.24 (0.86-1.79)	0.244

OR, odds ratio; CI, confidence interval. ^a^ χ^2^ test for genotype distributions between neuroblastoma cases and cancer-free controls. ^b^ Adjusted for age and gender. ^c^ Risk genotypes were rs1052536 TT and rs4796030 AA/AC.

**Table 2:** Stratification analysis of *LIG3* gene polymorphisms with neuroblastoma susceptibility

Variables	rs1052536 (cases/controls)		AOR (95% CI) ^a^	*P*^a^	rs4796030 (cases/controls)		AOR (95% CI) ^a^	*P* ^a^	Risk genotypes (cases/controls)		AOR (95% CI) ^a^	*P* ^a^
	CC/CT	TT			AA/AC	CC			0	1-2		
**Age, month**												
≤18	158/350	11/40	0.60 (0.30-1.20)	0.151	140/311	29/79	0.81 (0.50-1.29)	0.366	18/40	151/350	0.97 (0.54-1.75)	0.920
>18	265/555	35/53	1.38 (0.88-2.17)	0.162	238/479	62/129	0.96 (0.68-1.34)	0.792	27/76	273/532	1.48 (0.93-2.35)	0.101
**Gender**												
Females	178/376	18/38	1.00 (0.56-1.80)	1.000	157/323	39/91	0.88 (0.58-1.34)	0.551	21/53	175/361	1.23 (0.72-2.10)	0.455
Males	245/529	28/55	1.10 (0.68-1.78)	0.697	221/467	52/117	0.94 (0.66-1.36)	0.751	24/63	249/521	1.25 (0.76-2.04)	0.382
**Sites of origin**												
Adrenal gland	143/905	19/93	1.31 (0.77-2.22)	0.314	132/790	30/208	0.87 (0.57-1.33)	0.515	11/116	151/882	1.81 (0.95-3.44)	0.072
Retroperitoneal	126/905	12/93	0.91 (0.49-1.71)	0.773	111/790	27/208	0.93 (0.59-1.45)	0.743	15/116	123/882	1.06 (0.60-1.87)	0.846
Mediastinum	113/905	8/93	0.69 (0.33-1.46)	0.329	96/790	25/208	0.98 (0.61-1.56)	0.923	17/116	104/882	0.82 (0.47-1.42)	0.475
Others	34/905	6/93	1.68 (0.69-4.11)	0.256	33/790	7/208	0.80 (0.35-1.84)	0.599	1/116	39/882	5.09 (0.69-37.39)	0.110
**Clinical stages**												
I+II+4s	216/905	17/93	0.76 (0.44-1.30)	0.320	195/790	38/208	0.74 (0.50-1.08)	0.114	21/116	212/882	1.33 (0.82-2.17)	0.249
III+IV	189/905	27/93	1.40 (0.88-2.22)	0.151	165/790	51/208	1.18 (0.83-1.68)	0.359	24/116	192/882	1.05 (0.66-1.68)	0.839

AOR, adjusted odds ratio; CI, confidence interval. ^a^ Adjusted for age and gender, omitting the corresponding stratification factor.

## Abbreviations

NHEJ: non-homologous end-joining
*LIG3*: *Ligase III*
SNP: single nucleotide polymorphism
DSB: DNA double-strand break
N-Myc: N-myc proto-oncogene protein
