# Supplementary material for: Lack of associations between LIG3 gene polymorphisms and neuroblastoma susceptibility in Chinese children
Source: J Cancer. 2019 Oct 3;10(23):5722–6. doi: 10.7150/jca.33605 (PMC6843871; doi:10.7150/jca.33605)
Supplement: Supplementary file 1 — Supplementary figures and tables. [file jcav10p5722s1.pdf]

Supplemental Table 1. Frequency distribution of selected characteristics in cases and controls

Variables	Combined subjects					Shaanxi province				
	Cases (n=469)		Controls (n=998)		*P* [a]	Cases (n=76)		Controls (n=186)		*P* [a]
	No.	%	No.	%		No.	%	No.	%	
Age range, month	0.00-132.00		0.03-156.00		0.263	0.07-89.00		0.03-60.00		0.110
Mean ± SD	34.07±27.60		32.89±27.43			24.48±25.23		23.66±16.66		
≤18	169	36.03	390	39.08		43	56.58	85	45.70	
>18	300	63.97	608	60.92		33	43.42	101	54.30	
Gender					0.911					0.778
Female	196	41.79	414	41.48		28	36.84	72	38.71	
Male	273	58.21	584	58.52		48	63.16	114	61.29	
INSS stages										
I	133	28.42	/	/		64	84.21	/	/	
II	100	21.37	/	/		7	9.21	/	/	
III	69	14.74	/	/		1	1.32	/	/	
IV	147	31.41	/	/		4	5.26	/	/	
4s	11	2.35	/	/		/	/	/	/	
NA	8	1.71	/	/		/	/	/	/	
Sites of origin										
Adrenal gland	162	34.54	/	/		9	11.84	/	/	
Retroperitoneal region	138	29.42	/	/		51	67.11	/	/	
Mediastinum	121	25.80	/	/		12	15.79	/	/	
Other region	40	8.53	/	/		4	5.26	/	/	
NA	8	1.71	/	/		/	/	/	/	

SD, standard deviation; NA, not available.

[a] Two-sided χ^2 test for distributions between neuroblastoma cases and cancer-free controls.
